# Three-Dimensional Conformal Radiotherapy for Hepatocellular Carcinoma in Patients Unfit for Resection, Ablation, or Chemotherapy: A Retrospective Study

**DOI:** 10.1155/2013/780141

**Published:** 2013-11-28

**Authors:** Vassilis Kouloulias, Eftychia Mosa, John Georgakopoulos, Kalliopi Platoni, Ilias Brountzos, Anna Zygogianni, Christos Antypas, Paraskevas Kosmidis, Kyriaki Mystakidou, Maria Tolia, Ivelina Beli, Athanasios Gouliamos, John Kouvaris, Nikoalos Kelekis

**Affiliations:** ^1^Radiotherapy Unit, 2nd Department of Radiology, ATTIKON University Hospital, Medical School, Athens, Rimini 1, 12462 Xaidari, Greece; ^2^Radiotherapy Unit, 1st Department of Radiology, Aretaieion University Hospital, Medical School, Athens, Greece; ^3^Medical Oncology Unit, Ygeia Hospital, Athens, Greece

## Abstract

*Purpose*. The purpose is to evaluate the feasibility, efficacy, and the toxicity of three-dimensional conformal radiotherapy (3DCRT) in patients with advanced hepatocelluar carcinoma (HCC) and inferior vena cava tumor thrombosis (IVCTT). *Methods*. Between 2007 and 2012, in a retrospective way, 9 patients (median age 69 years) with advanced HCC and IVCTT unfit for surgery, radiofrequency ablation, embolization, or chemotherapy were treated with three-dimensional conformal radiotherapy (3DCRT). The radiotherapy volume included both primary tumor and IVTT. The radiotherapy schedule was 50–52 Gy in 2 Gy fractions. Overall survival (OS), response to radiotherapy, visual analogue scale (VAS), and toxicity were assessed. *Results*. All patients demonstrated a response rate up to 60%. During radiotherapy, 3 patients experienced grade 1 nausea/vomit toxicity. All patients demonstrated an elevation of the liver enzymes (3 patients with grade 1 and 6 patients with grade 2). The mean VAS-score was decreased from 6.11 to 3.11, while the median overall survival was 24 months. *Conclusion*. 3DCRT achieves a very high local control rate and is suitable for patients with HCC and IVTT, while the documented radiation induced toxicity is moderate. It can be recommended for palliation in patients unable to undergo curative therapies.

## 1. Introduction

Hepatocellular carcinoma (HCC) is the fifth most common malignancy worldwide and the third most common cause of cancer-related death, following lung cancer and stomach cancer in the whole world [1].

Patients suffering from chronic Hepatitis B have a tendency in appearing HCC. Cirrhosis, Hepatitis C and aflatoxin B exposure are also other usual risk factors [2].

Partial hepatectomy is the treatment of choice for patients with good liver function and resectable tumors. According to Milan criteria, liver transplantation can offer cure to people with advanced cirrhosis and tumors smaller than 5 cm in greatest dimension, or up to 3 tumors each smaller than 3 cm, with no extrahepatic spread and without vascular invasion [3].

For patients who are not surgical candidates but have tumors smaller than 3 to 4 cm in size, percutaneous ablation, or radiofrequency ablation (RF) can offer long term control. RF represents the placement of a needle in the tumor, percutaneously or laparoscopically, under image guidance, so that a high frequency alternating current can heat the surrounding tissue to produce necrosis. The local recurrence rate is less than 10%. However, lesions near the dome of the liver are less suited for RF since they are less well visualized by ultrasound while lesions near large vessels cannot be adequately heated because of the heat sink from the adjacent blood flow.

Transcatheter arterial chemoembolization (TACE) is an alternative treatment modality for HCC. It is known that HCC derives 80% of its blood supply from the hepatic artery whereas the normal liver parenchyma is supplied by the portal vein. TACE exploits HCC's preferential blood supply from hepatic artery to deliver chemotherapy without damaging the surrounding liver parenchyma. The intended purpose of embolization is to prevent the washout of the drug at the site of tumor and to induce ischemic necrosis. Usually the injection of the embolic particles follows the injection of the chemotherapeutic mixture. Because of that information, it is explained why TACE can target HCC with specificity [4–6]. The best candidates for TACE are patients with unresectable and asymptomatic lesions, with preserved liver function and without vascular invasion or extrahepatic spread. The problem arises from the coexistence of inferior vena cava thrombosis (IVTT) and/or portal vein tumor thrombosis (PVTT). In these particular situations, TACE shows a lack of efficacy and a high risk of ischemic liver insufficiency when performed.

Thrombosis of the large vessels is associated with portal vein hypertension, deterioration of the liver function, and tumor dissemination. These symptoms reduce the possibility of surgical resection, or transarterial chemoembolization on HCC. IVTT and PVTT are common complications in patients with advanced HCC. When tumor invades the intrahepatic large vessels, the prognosis is poor and if no treatment is applied, the median survival of patients with HCC and IVTT is three months [7]. The recurrence of HCC and the rapid development of metastasis are common in these patients.

Sorafenib, is a multitargeted anti-VEGF receptor and a raf kinase inhibitor that is approved for the treatment of unresectable HCC. Depending on the etiology and the extent of cirrhosis, the outcomes of the systemic therapy varies according to the relative literature [8].

Another treatment option for patients with HCC is radioembolization through Yttrium microsphere therapy. It can be performed in patients that are candidates for orthotopic liver transplantation (OLT), in patients unfit to undergo resection but without PVTT and in patients with advance disease. Unlike radiofrequency ablation that has a limited role due to the risk of tract seeding and challenges related to size and location of tumors, radioembolization has been shown to abort the progression of the disease, which can allow the patients more time to wait for donor organs. Thus, radioembolization succeeds bridging and downstaging potential transplant candidates, as well as it can be used as a palliative modality in patients with multifocal disease, particularly those with vascular invasion. Potential advantages over TACE include fewer treatment sessions and the ability of outpatient treatment basis.

According to Lau et al. [9], a single dose administration of intra-arterial ^131^I-lipiodol given, after curative resection of HCC, could significantly decrease the recurrence rate and could increase disease-free survival and overall survival. Based on the available evidence, intra-arterial ^131^I-lipiodol therapy was also found safe and effective, as a palliative modality for unresectable HCC. The radiation activity that is released from this radionuclide after an intra-arterial injection of 1850 MBq ^131^I-lipiodol is about 4463 cGy. The drawback of this modality is that the patients have to be isolated for radiation protection, until the activity of ^131^I is below 370 MBq. That means that they have to remain isolated for about 10–14 days, depending on the effective half-time of ^131^I-lipiodol. Additionally, before, during and after the therapy, the thyroid gland has to be protected by the uptake of free ^131^I [10].

All the above treatment options can be used in patients unfit to undergo resection or liver transplantation. However, when the patients suffer from advanced HCC with IVTT and cirrhosis, TACE, sorafenib, and radioembolization cannot be performed with safety. Since there is no standard therapy for patients with cirrhosis, HCC and IVTT, three-dimensional conformal radiotherapy can be considered as an option.

The development of 3DCRT has enabled high dose RT to be directed to the tumor while at the same time can spare the non-tumor bearing surrounding liver parenchyma, from these high doses. Using a mathematical model that predicts the risk of radiation induced liver disease (RILD), the probability of radiation toxicity can be minimized. The aim of our study is to investigate the potential efficacy together with the acute toxicity in patients with cirrhosis, advanced HCC, and IVTT that are unfit for resection, ablation, or chemotherapy.

## 2. Materials and Methods

We are reporting our results from a retrospective study of selective patients with advanced HCC and IVTT that were not eligible for surgery, radiofrequency ablation, embolization, or chemotherapy that were treated with three-dimensional conformal radiotherapy (3DCRT).

Between 2007 and 2012, in a retrospective way, 9 patients that had diagnosed with advanced HCC and IVTT received 3DCRT. All the candidates were men and suffered from hepatitis B, had cirrhosis and showed inferior vena cava thrombosis. The median age was 69 years old. The patients' characteristics are shown in Table 1.

The pretreatment evaluation included pathology review with risk factors. A diagnosis of HCC was made either histologically or based on typical radiological findings of HCC using two dynamic imaging studies (CT, magnetic resonance imaging, or hepatic angiography), or one positive finding with an elevated serum alpha-fetoprotein (AFP) level >200 ng/mL. All patients had undergone laboratory studies with complete blood count, chemistries, coagulation panel, serum AFP, and Hepatitis B/C panel. Patients were staged using TNM classification system [11].

The inclusion criteria were as follows: HCC stage T2–T4 with IVTT, unresectable disease or medically unsuitable for resection, more than 800 cc of uninvolved liver, Eastern Cooperative Oncology Group Performance status 0–2 and Child-Pugh Stage A-B. Exclusion criteria were as follows: previous RT to the abdomen, Child-Pugh C cirrhosis, ongoing immunosuppressive therapy and patients who initially presented with multiple intrahepatic metastases in both lobes.

All patients were required to sign an informed consent form previously, concerning the side effects of irradiation. For treatment planning purposes, each patient underwent a virtual CT-simulation, in supine position with customized immobilization device. Bilateral arms were abducted and externally rotated. Contrast-enhanced computed tomography scan was acquired at 3 mm slice thickness, during quiet breathing. The CT datasets were transferred either to Prosoma or ONCENTRA Virtual simulation and contouring system through the DICOM network. All contouring of target volumes and normal structures (organs at risk-OARs) were performed. The following structures were delineated: gross tumor volume (GTV), clinical target volume (CTV), and planning target volume (PTV) according to the ICRU criteria [12, 13]. Organs at risk (OARs) included normal liver, the kidneys, the stomach, the small intestine, and the spinal cord. Normal liver volume was defined as the total liver volume minus the GTV.

Patients were treated on a supine position with the arms above the head on a wing board. The gross tumor volume (GTV) included both primary tumor and IVTT as they were visualized on the planning CT or after fusion with magnetic resonance imaging. The clinical target volume (CTV) was defined as the GTV plus a 1 cm margin in all directions. The planning target volume (PTV) was defined by adding a 0.5 cm margin for set-up error and for compensation of the organs' movement during normal breathing.

Based on baseline liver function and liver volume receiving ≥20 Gy, the radiotherapy schedule that delivered was 50–52 Gy in 2 Gy fractions and was prescribed to the planning target volume surface. The selection of the total dose was related to the dose distributions and when necessary the total dose was prescribed to 50 Gy from 52 Gy. The treatment planning consisted of 4 beams. All the fields were treated with either 6 MV or 15 MV photon beams. The target dose of 2 Gy per fraction was administered daily and was prescribed to 95% at the International Commission on Radiation Units and Measurements reference point, at the intersection of the central axis of the treated beam in the midplane of the target volume. A total dose of 50–52 Gy was prescribed. All patients were treated on either a VARIAN CLINAC 2100C or a SIEMENS Oncord linear accelerator. During radiotherapy treatment course, all patients were under antiemetic treatment, for the prevention of symptoms like nausea and vomiting.

The subject of this study was to assess the efficacy, feasibility, and toxicity of 3DCRT in patients with HCC and IVTT.

### 2.1. Endpoints

Endpoints included treatment feasibility, toxicity outcomes, and short-term efficacy. Treatment feasibility was defined as the successful delivery of the prescribed dose following the intended treatment schedule. Efficacy was based on the rate of local recurrence and overall survival.

The images were systematically reviewed by an expert who classified responses as partial, complete, or progression based on vascularization according to the European Association for the study of the Liver (EASL) criteria [14] and the Response Evaluation Criteria in Solid Tumors (RESIST) [15].

Radiation Induced Liver Toxicity was graded according to EORTC/RTOG toxicity criteria [16].

Patients were evaluated weekly during radiotherapy. The protocol recommendation was to check alkaline phosphatase, transaminase levels, bilirubin and the symptom of pain, through the visual analogue scale (VAS) during and after the radiotherapy [17]. All patients were reviewed every month later on, after the completion of the radiotherapy, in order to assess toxicity.

Data at diagnosis (baseline), at the end of radiation treatment, and from all monthly follow up visits, were evaluated. The response to treatment was assessed 4 months after radiotherapy, by analyzing the tumor dimensions at *x*, *y*, and *z* axis. Symptoms occurring in the interval between the start of radiotherapy and 90 days after this time point were classified as “acute”. Toxicity grading followed the EORTC/RTOG criteria [16].

To evaluate the dose constraints for normal tissues we used the QUANTEC trial [18–24].

### 2.2. Statistical Analysis

The comparison of the three dimensions of the lesions (at *x*, *y*, *z* axis) and VAS score at baseline and 4 months post RT was performed with the Wilcoxon non-parametric test. The survival analysis was done with the Kaplan-Meier method. The significance level was set at 0.05. The whole analysis was performed with SPSS ver 10 software (IL, USA).

## 3. Results

**Figure 1:**
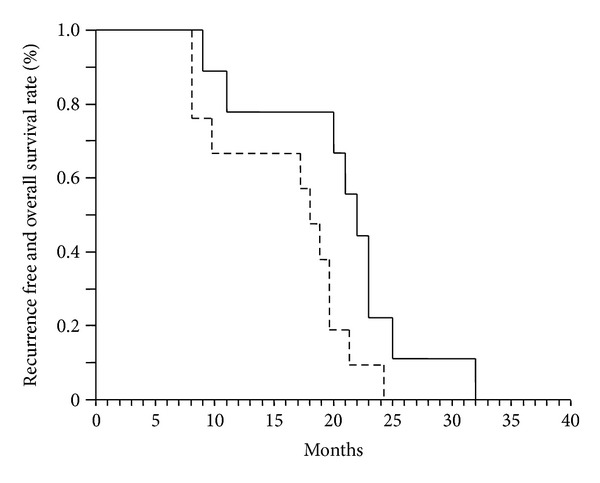
Kaplan Meier curve of recurrence free (dotted line) and overall (solid line) survival (median 21 and 22 months, resp.).

All patients according to Eastern Cooperative Oncology Group had performance score of ≤2 and Child-Pugh score from A5–B9. The median recurrence free and overall survival was 21 (SE = 4) and 24 (SE = 4) months, respectively (Figure 1). All patients died from local progressive disease along with bone metastases.

No radiation-induced liver disease was observed. The mean values of *x*, *y*, *z* axis and VAS-score before and 4 months post RT were decreased significantly (*P* < 0.001, Wilcoxon test).

**Figure 2:**
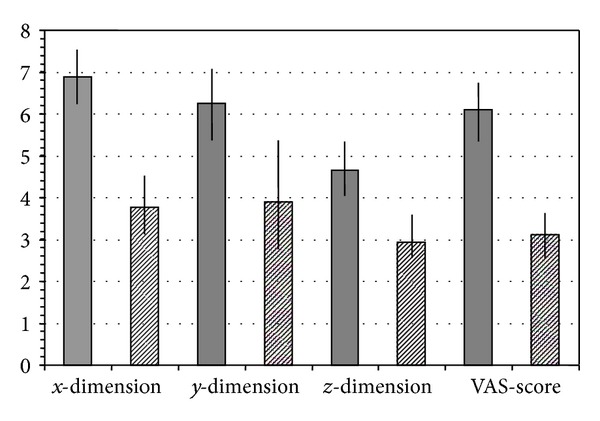
Mena values for the dimensions in *x*, *y*, and *z* axis of the primary site and VAS score before and after RT.  *P* < 0.001, Wilcoxon test. Vertical lines stand for ±SD.

At baseline, the mean values of *x*, *y*, *z* dimensions were 6.878 cm (SD = 0.845), 6.256 cm (SD = 1.114), and 4.656 cm (SD = 0.791) respectively, before RT. The dimensions of the lesions post RT, reduced to *x* = 3.76 cm (SD = 1.04), *y* = 3.889 cm (SD = 1.367), *z* = 2.994 cm (SD = 0.573). Additionally, the VAS-score before radiotherapy was 6.11 (SD = 0.93), while post RT, the VAS-score modified to 3.11 (SD = 0.60) (Figure 2). The treatment response rate was approximately 60%.

Overall, the treatment was well tolerated. As shown in Table 2, no treatment-related grade 4 or 5 acute toxicity was seen within 3 months after 3DCRT. Grade 1 nausea/vomiting was not a common toxicity encountered during radiotherapy, while 3 patients had symptoms. All patients experienced elevation of liver enzymes (3 patients with Grade 1 and 6 with Grade 2). Seven patients experienced Grade 2 decrease in their platelets.

## 4. Discussion

Patients with HCC, IVTT, and cirrhosis are not eligible to undergo resection or liver transplantation. Traditionally, HCC has been considered a radio-resistant tumor because the dose delivered by conventional external beam radiotherapy could not exceed 30 Gy on the whole liver due to the risk of fatal radiation induced hepatitis.

Radiation induced liver disease (RILD) is a syndrome that appears due to normal tissue complications. According to Kim et al., RILD is often called radiation hepatitis and is characterized by the development of nonmalignant ascites without disease progression. There is an anicteric increase in the alkaline phosphatase level of at least twofold or in the transaminase level of at least fivefold and this occurs 2 weeks to 4 months after liver radiotherapy. Risk factors for RILD are the existence of hepatitis B carrier status and Child-Pugh B. Dosimetric variables suggested to be associated with an increased risk of nonclassic RILD include volume receiving ≥20 Gy (V20) [25].

On the one hand, most of the trials using conventional doses of radiation resulted in objective response rates of approximately 15% and toxicity rates >80% with low 6-month recurrence-free survival (33%) [26].

On the other hand, HCC was found to be radiosensitive for radiation doses >30 Gy. With the advanced technology of 3DCRT, the target can be of limited volume, well conformed to tumor, sparing the surrounding normal liver parenchyma and limiting radiation-induced side effects [27]. The development of 3DCRT has enabled high dose radiotherapy to be directed to the tumor while sparing the non-tumor surrounding liver parenchyma from these high doses. More specifically, when 3DCRT is used, it is safe to target only the tumor and the IVTT, plus the closely abutting partial volume of hepatic tumor.

Data from Taiwan cohort including 44 patients with advanced HCC (6–25 cm) confirmed the feasibility and efficacy of 3DCRT, delivering a total dose of radiotherapy ranging from 40–60 Gy in cirrhotic patients. In this study, overall survival rate improvement correlated with the total dose delivered to the tumor, especially for doses >50.4 Gy [28]. In our study it has been proved that there is a good correlation between the total dose of 50–52 Gy and the objective response since it was demonstrated a treatment response rate of up to 60%, in patients with HCC, IVTT, and cirrhosis unfit for other modalities.

Choi et al. investigated the prognostic significance of PVTT response in patients with HCC, when they were treated with concurrent chemoradiotherapy. They analyzed 100 patients that underwent radiotherapy of 45 Gy and they concluded that complete response of the PVTT was strongly correlated with improved survival. Additionally, when objective response was achieved in both the tumor and the PVTT, these were also associated with further improvement in survival [29]. Well promising results have been published too, when hypofractionated schemes were delivered to HCCs and IVTT through stereotactic body radiotherapy, demonstrating that liver is radiosensitive, the control of IVTT is and independent prognostic factor and that treatment response leads to better overall survivals [30, 31].

According to the study by Huang et al., 326 HCC patients with PVTT were treated with 3DCRT with a total dose of 60 Gy in 2-3 Gy fractions. The treatment responses were classified from the changes of the image on CT and Doppler ultrasonography by observing the regression of thrombi and the restoration of portal blow flow. The study showed that the most significant independent variable for survival was the performance status and a radiation dose more than 50 Gy. These studies are in accordance with the results from our trial that showed a median survival of 24 months, in patients of Child Pugh A-B that underwent radiotherapy to the dose of 50–52 Gy [32]. More specifically, it was achieved a median recurrence free survival and a median overall survival of 21 months and 24 months, respectively, unlike the expected three-month life, when no treatment is delivered to this group of patients with HCC and IVTT.

Mornex et al. studied 27 patients with Child-Pugh A/B cirrhosis with small size HCC, nonsuitable for curative treatments that had undergone radiotherapy. The results demonstrated that patients with well-compensated cirrhosis, Child-Pugh A, tolerate 3DCRT well [33]. Tanaka et al. confirmed that hepatic function of Child-Pugh was an independent factor for both RT responds and overall survival [34]. Our experience showed that all patients completed radiotherapy with minimal side effects, since nausea and vomit were not that common symptoms and the elevation of the hepatic enzymes were not differentiated a lot from the radiotherapy scheme. Additionally, grade 2 thrombocytopenia was noted in seven patients, but was not resistant and the level of the platelets became normal a little while after the end of the treatment.

According to recent studies, radiotherapy can be successfully combined with other treatment modalities, as TACE. Especially in patients with IVTT or PVTT, radiotherapy can be used as a treatment option in order to shrink the vascular thrombus. In that way, TACE can be more effective. According to Koo et al. the combination of radiotherapy and TACE had higher response rates (43% versus 13) and overall survival (median 11.7 versus 4.7 months) than TACE alone [35, 36].

Nevertheless, even when another modality has failed to control HCC, 3DCRT, is an alternative treatment option since it demonstrates good results. More specifically, Wigg et al. have published a case report, where conventional 3DCRT was used successfully, as a neoadjuvant treatment for downstaging HCC in order to follow liver transplantation. In that concrete case, the patient had undergone TACE unsuccessfully and there was a disease progression, beyond transplantable criteria. 3DCRT was performed (54 Gy, in 27 fractions) in order to downstage the lesion. There was a complete radiological response for 16 months and the liver transplantation took place. The final histopathological report showed complete response with necrosis, too. Consequently, 3DCRT may not only be an alternative treatment option, but shows promising results as a neoadjuvant modality, too [37].

## 5. Conclusions

Our study showed that 3DCRT is a feasible and safe modality to treat patients with HCC, IVTT and cirrhosis, with a scheme of 50–52 Gy in 2 Gy fractions. The encouraging treatment results have confirmed the clinical value of radiation therapy in those patients and can be recommended as a treatment option, while a prospective randomized study stands in need for the confirmation of our results.

## Figures and Tables

**Table 1:** Characteristic of patients, tumors, and IVTT.

Gender	
Male	9
Female	0
Age (years)	
Median	69
Range	59–77
Viral etiology	
HBsAg (+)	9
HBsAg (−)	0
AFP (ng/mL)	
≤400	2
≥400	7
Mean tumor dimensions (cm)	
*x*-axis	6.878
*y*-axis	6.256
*z*-axis	4.656
VAS score	6.11
Number of patients with IVTT	9

**Table 2:** Acute toxicity (*n* = 9 patients).

CTC	Grade 0	Grade 1	Grade 2	Grade 3–5
Nausea/vomiting	6	3	—	—
Liver enzymes	—	3	6	—
Bilirubin	9	—	—	—
Anemia	6	3	—	—
Leucocytes	7	2	—	—
Platelets	—	2	7	—

CTC: common toxicity criteria.
